# Microbial Composition and Functional Diversity Differ Across Urban Green Infrastructure Types

**DOI:** 10.3389/fmicb.2020.00912

**Published:** 2020-06-05

**Authors:** Aman S. Gill, Kai Purnell, Matthew I. Palmer, Jaime Stein, Krista L. McGuire

**Affiliations:** ^1^Department of Environmental Science and Policy Management, University of California, Berkeley, Berkeley, CA, United States; ^2^Department of Biology, Barnard College, New York, NY, United States; ^3^Department of Ecology, Evolution and Environmental Biology, Columbia University, New York, NY, United States; ^4^Programs for Sustainable Planning and Development, School of Architecture, Pratt Institute, Brooklyn, NY, United States; ^5^Department of Biology, Institute of Ecology and Evolution, University of Oregon, Eugene, OR, United States

**Keywords:** microbial ecology, community assembly, functional diversity, metagenomics, microbial biogeography, urban ecology, ecosystem services, green infrastructure

## Abstract

Functional and biogeographical properties of soil microbial communities in urban ecosystems are poorly understood despite their role in metabolic processes underlying valuable ecosystem services. The worldwide emergence of engineered habitats in urban landscapes—green roofs, bioswales, and other types of soil-based green infrastructure—highlights the importance of understanding how environmental changes affect the community assembly processes that shape urban microbial diversity and function. In this study we investigated (1) whether engineered green roofs and bioswales in New York City had distinct microbial community composition and trait-associated diversity compared to non-engineered soils in parks and tree pits, and (2) if these patterns were consistent with divergent community assembly processes associated with engineered specifications of green infrastructure habitats not present in conventional, non-engineered green infrastructure; specifically, tree pit and park lawn soils. We found that green roofs and bioswales each had distinct bacterial and fungal communities, but that community composition and diversity were not significantly associated with geographic distance, suggesting that the processes structuring these differences are related to aspects of the habitats themselves. Bioswales, and to a lesser extent green roofs, also contained increased functional potential compared to conventional GI soils, based on the diversity and abundance of taxa associated with nitrogen cycling, biodegradation, decomposition, and traits positively associated with plant growth. We discuss these results in the context of community assembly theory, concluding that urban soil microbial community composition and diversity in engineered habitats are driven largely by environmental filtering, whereas stochastic processes are more important among non-engineered soils.

## Introduction

Understanding the ecological dynamics of urban soil microbiomes is an increasingly practical matter as municipalities expand green infrastructure (GI) programs featuring engineered green spaces designed to restore and enhance ecosystem services (Tzoulas et al., 2007). A leading example is New York City’s multi-billion dollar Green Infrastructure Plan to use bioswales, green roofs, and other engineered habitats to absorb and filter stormwater runoff, reducing the need for costly sewer retrofitting and expanded wastewater processing (NYC Department of Environmental Protection [DEP], 2010a, b). Although the primary design and monitoring focus is on hydrology, GI installations are also expected to generate co-benefits like biodiversity support and mitigation of urban heat island effects (NYC Department of Environmental Protection [DEP], 2017). Additional ecosystem services provided primarily by soil bacteria and fungi include nutrient assimilation and cycling, bioremediation of wastewater contaminants, and decomposition (Morel et al., 2015). Prospects for incorporating the management of microbial biodiversity and ecosystem services into GI designs, although promising, are presently limited by our incomplete understanding of how anthropogenically-controlled factors shape fine-scale patterns of soil microbial diversity and function across urban landscapes.

Existing studies establish that urban soil microbial communities host surprisingly diverse bacterial and fungal communities despite persistent, acute stresses including dense human traffic, impervious surfaces, heat island effects, pollution, and resource fluxes. Research on Central Park (Ramirez et al., 2014), medians (Reese et al., 2016), and human-altered soils (Huot et al., 2017) in NYC indicate that soil microbial composition is related to microsite differences, especially in relation to edaphic characteristics like pH, nutrient content, and plant associations. It remains unclear how site specific differences in GI installations and other urban soil types contribute to biogeographical patterns in the distribution of urban soil microbial taxa. Bioswales, green roofs, and other GI environments experience the same stressors that afflict parks, medians, tree pits, and other fragmented urban soil micro-habitats. Yet GI sites are also explicitly designed to exhibit unique environmental characteristics that could help shape microbial community diversity, composition, and function. GI installations are filled with engineered Technosol soils (FAO of the UN, 2015) with specified particle size range and composition, nutrient content, and pH (NYC Parks Department, personal communication). Compared to other urban soils, GI sites also have proscribed plant communities, resource and water fluxes associated with stormwater intake, protection from pedestrian traffic, and ongoing monitoring and maintenance (NYC Department of Environmental Protection [DEP], 2017). Despite their proximity to heavily used roads, GI soils have relatively low levels of metal and petroleum-derived hydrocarbon contamination (Deeb et al., 2018). There is some evidence to suggest that microbes in GI installations are compositionally distinct, as seen for fungal communities in NYC green roofs compared to city parks (McGuire et al., 2013), and among bacterial communities in different types of bioswales (Joyner et al., 2019).

Major shifts in belowground communities are likely to be consequential since microbes drive fundamental chemical and energy transformations, including several pertaining directly to key ecosystem services that are valuable in urban contexts (King, 2014). For example, numerous nitrogen cycling processes are carried out by diverse bacterial lineages, including denitrification of nitrate to nitrogen gases (Groffman and Crawford, 2003). Stormwater contains high concentrations of reactive nitrogen that can be assimilated by soil bacteria, reducing eutrophication in surrounding waters (Groffman et al., 2002; Taylor et al., 2005; Hale et al., 2014). Stormwater runoff also carries petroleum-derived hydrocarbons, pesticides, and heavy metals (Makepeace et al., 1995), highlighting potential bioremediation properties of microbes that can metabolize these substrates. Fungal and bacterial decomposition of organic matter is an important regulator of energy and matter in all ecosystems, including cities (Ossola et al., 2016). Determining the diversity and distribution of microbes associated with these and other stormwater-related functions is an important step in assessing microbially-provisioned ecosystem services in GI installations (Gill et al., 2017).

Ultimately, managing urban soil microbiomes will require a predictive understanding of the ecological and evolutionary processes that mediate how anthropogenic and environmental factors shape microbial community assembly and function. The various factors influencing community assembly can be grouped into four categories: evolutionary diversification (speciation and extinction), stochastic forces like drift, dispersal or migration, and ecological selection, i.e., biotic or abiotic factors that influence the availability and competition for niche space (Vellend, 2010). All four operate simultaneously and at multiple temporal and spatial scales, but are unlikely to have equivalent importance at local scales, where ecological factors tend to act faster than processes rooted in history or regional biogeography (Ricklefs, 2004). Urban microbial communities are likely shaped by environmental filtering of regional species pools based on compaction, pollution, nutrient loading, and other ubiquitous stressors associated with urbanized ecosystems (Shochat et al., 2006). The balance of stochastic processes and deterministic, niche-based forces across urban habitats remains unclear (Morrison-Whittle and Goddard, 2015). Bacterial communities in medians and parks were undifferentiated in one study (Reese et al., 2016), consistent with neutral community assembly. On the other hand, the unique designs and resource inputs of engineered GI habitats described above may result in additional environmental filtering and species sorting, resulting in deterministic impacts on microbial diversity relative to GI sites with non-engineered soils.

In this study we used 16S and ITS amplicon sequencing to analyze patterns of bacterial and fungal community composition in a survey of engineered GI habitats (bioswales and green roofs) and non-engineered or conventional GI soils (tree pits and park lawns) co-distributed across all five boroughs of New York City. We also examined functional diversity by analyzing the composition and diversity of bacterial genera linked to nitrogen cycling, decomposition and biodegradation traits, as well as saprotrophs and other fungal guilds. We use these data to address three hypotheses: (1) GI bioswales and green roofs are compositionally distinct from conventional, non-engineered GI soils. (2) GI design specifications contribute to deterministic processes in engineered GI sites that result in distinct, non-neutral differences in community assembly compared to conventional GI soils. (3) Bioswales, green roofs, and conventional, non-engineered tree pits and park lawns have distinct compositions of functional taxa linked to nitrogen cycling, bioremediation, decomposition, and fungal guilds.

## Materials and Methods

### Site Selection and Sample Collection

**FIGURE 1:**
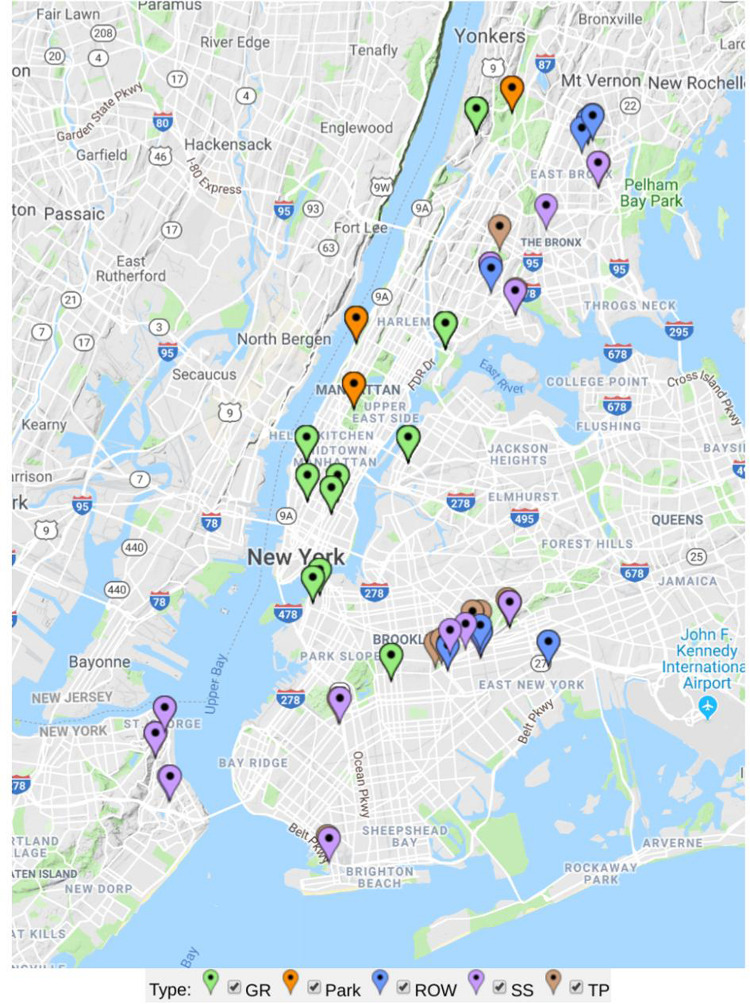
Map of sample sites analyzed in this study separately colored by green infrastructure type (GR in green = green roof, Park in orange = ground-level park lawns, ROW in blue = right-of-way bioswale, SS in purple = streetside infiltration swale, and TP in brown = conventional tree pit).

The sample sites in the study were selected from the five boroughs of New York City (Figure 1). The city lies between 40.6–40.9° N, 73.8–73.9° W, 0–50 m above sea level. New York City has a mean annual precipitation of 1142 mm, and had a mean annual temperature of 13.1°C in 2013 (National Oceanic and Atmospheric Administration, 2015). All samples were taken between July and October of 2013.

Sites included a distribution of engineered green infrastructure installations (bioswales and green roofs) and non-engineered tree pits and park lawns. Although all urban sites are engineered in the sense that they are human-made habitats, green roofs and bioswales constructed as part of NYC’s Green Infrastructure Plan use standard designs with specified substrate particle size, pH, nutrient content, plant communities, management protocols, infrastructural functions, and many other attributes (NYC Department of Environmental Protection [DEP], 2017). We sampled tree pits and park lawns as conventional, non-engineered GI soils control sites not constructed or maintained according to the same guidelines, allowing us to address whether GI sites have distinct microbial composition within the broader landscape of urban microhabitats. Engineered GI sites included green roofs and two types of bioswales: relatively small right-of-way swales (5–6 feet wide by 10–20 feet long) built into pedestrian sidewalks, and larger streetside swales often occupying a corner or median area not along pedestrian throughways (NYC Department of Environmental Protection [DEP], 2017). Thirty-four of the sites were conventional tree pits, 13 were right-of-way (ROW) bioswales, 12 were streetside (SS) infiltration swales (also called stormwater greenstreets), 3 were park lawns, and 11 were green roofs. Soil or growing media was sampled from each site using a soil corer 2.5 cm in diameter. The corer was cleaned with ethanol between collection of each sample. Cores were stored in sterile Whirl-Pak bags (Nasco, United States), and frozen at −20°C after being stored in a cooler for 2–4 h from the time of sampling. Four 10 cm cores were taken for each sample. One sample was taken from each conventional tree pit (roughly 4 × 5 ft.), ROW bioswale (5 ft. wide by 10, 15, or 20 ft. long), and SS swales which can be two to three times larger than ROW swales (NYC Department of Environmental Protection [DEP], 2017). Three to ten samples were taken from much larger park lawns and green roofs—for each of these samples cores were taken from four contiguous 1 m × 3 m areas arranged as a grid.

### Microbial Community Analyses

Soil was sieved for homogenization with UV-sterilized 2 mm sieves. DNA was extracted from 0.25 g of each homogenized soil sample using the PowerSoil-htp 96-Well Soil DNA Isolation Kit and PowerSoil DNA Isolation Kit, according to the manufacturer’s directions, with an additional incubation step at 65°C for 10 min followed by 2 min of bead beating (Mo Bio Laboratories, Inc., Carlsbad, CA, United States).

The first internal transcribed spacer region (ITS1) of fungal rDNA (ribosomal DNA) was amplified using the ITS1-F (CTTGGTCATTTAGAGGAAGTAA) and ITS2 (GCTGCGTTCTTCATCGATGC) primer pair (Gardes and Bruns, 1993; Bellemain et al., 2010). For bacterial analyses, the V4 hypervariable region of 16S rDNA was amplified with a 515-F (GTGCCAGCMGCCGCGGTAA) and 806-R (GGACTACHVGGGTWTCTAAT) primer pair (Caporaso et al., 2012). Illumina adapters, primer pad, and 2-bp linker sequences were affixed to both forward and reverse primers, and the reverse primer contained a 12-bp error-correcting barcode that was specific to each sample.

DNA was amplified twice per sample in PCR reactions of 13 μL water, 10 μL 5-Prime Hot Master Mix, 0.5 μL each of the forward and reverse primers, and 1.0 μL DNA (McGuire et al., 2013). Reactions were held at 94°C for 3 min, followed by 35 cycles at 94°C for 45 s, 50°C for 60 s, and 72°C for 90 s, with a final extension of 10 min at 72°C. PCR products were visualized on 1.2% agarose gel.

High-throughput Illumina sequencing was used to survey the diversity and composition of the fungal and bacterial communities found in each sample. Amplicon concentrations were quantified with PicoGreen dsDNA assay, and amplicons from all samples were pooled in equimolar volumes before sequencing using an Illumina Miseq instrument at the New York Medical Center Genomics Core Laboratory in Valhalla, New York.

### Sequence Processing

Amplicon reads for the 16S V4 region were de-multiplexed with deML^1^ and processed using DADA2, including quality filtering with maxEE = 2 (Callahan et al., 2016). A read error model was parameterized from the data and used to resolve dereplicated reads into unique 16S amplicon sequence variants (herearfter referred to as ASVs or simply “variants”). Paired-end reads were then merged and mapped to ASVs to construct a sequence table, followed by removal of chimeric sequences. Taxonomic assignments were made using exact matches of ASVs and reference strains from the Ribosomal Database Project database (Cole et al., 2009). Sequence tables and taxonomic assignments were imported into R for all downstream processing and analysis (R Core Team, 2019). To account for variation in sequencing effort across samples, samples were scaled according to variance stabilized ASV abundances using DESeq2 (Love et al., 2014). 16S ASVs were aligned with DECIPHER (Wright, 2016) and used to infer a neighbor-joining tree, which was then used as the starting point to derive a maximum likelihood tree with a generalized time-reversible model with gamma rate variation, implemented with the phanghorn package in R (Schliep, 2011). A similar process was used for ITS reads, except QIIME was used for demultiplexing (Caporaso et al., 2010), adapters and primers were trimmed with cutadapt (Martin, 2011), taxonomic assignments were based on the UNITE database (Nilsson et al., 2019), and no tree was inferred due to the variable length of the ITS region.

### Trait-Associated Taxa

To assess the functional potential of microbial communities we relied on published studies with manually curated lists of bacterial genera associated with specific metabolic processes. Nitrogen cycling taxa representing eight pathways—denitrification, assimilatory and dissimilatory nitrate reduction, N fixation, ammonia assimilation, and assimilatory and dissimilatory nitrite reduction—were compiled based on Nelson et al. (2016). Bacterial genera linked to decomposition pathways were compiled based on multiple surveys of decomposition processes in multiple habitat types (Eichorst and Kuske, 2012; Štursová et al., 2012; Yang et al., 2014; López-Mondéjar et al., 2016). A list of genera associated with biodegradation of petroleum-derived hydrocarbons, diverse aromatic compounds, and various pesticides was identified based on manual searches of the Biocatalysis/Biodegradation Database (Gao et al., 2010). Fungal guilds were identified using FUNGuild v1.1 (Nguyen et al., 2016). Functional diversity estimates based on these genus-level lists represent considerable taxonomic and metabolic diversity, but are also limited due to the incomplete nature of data sources and ambiguity of taxonomic identifications based on a subset of 16S rDNA variable regions. For simplicity, we refer to the diversity of these subsets of taxa as “functional diversity,” recognizing this metric does not directly estimate “true” or total functional diversity in these communities.

### Data Analyses

Compositional differences across green infrastructure types, soil types (GI vs. non-engineered GI), and boroughs were evaluated using Analysis of Similarity tests and Bray–Curtis metrics in R using the vegan package (Oksanen et al., 2017). Alpha diversity metrics were likewise calculated using the vegan package. Relative abundances of ASVs were square-root-transformed prior to these analyses. Non-metric multidimenensional scaling (NMDS) plots were used to visualize possible clustering patterns in communities. To examine patterns of phylogenetic clustering or over-dispersion we calculated the mean pairwise distance (MPD) for all pairs of genera using the ‘picante’ package in R (Kembel et al., 2010). The calculated MPD was compared to a null model for phylogenetic dispersion based on shuffling distance matrix labels using the function ses.mpd.

## Results

### Bacteria

**FIGURE 2:**
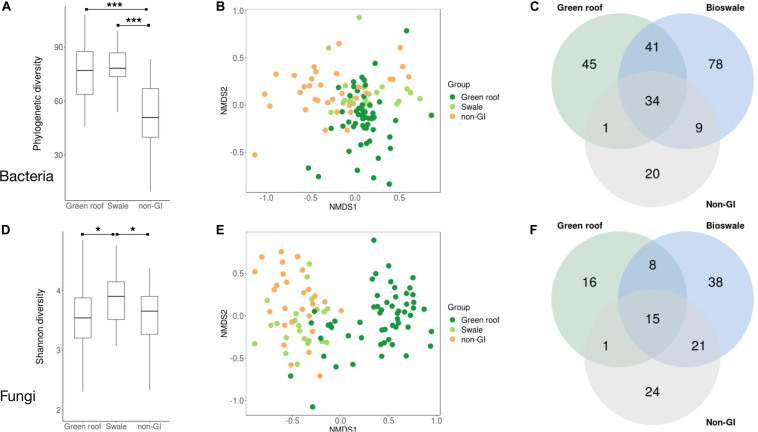
Summary of diversity metrics for bacterial **(A–C)** and fungal **(D–F)** communities. Phylogenetic diversity **(A,D)**, NMDS ordination plots based on Bray–Curtis distances **(B,E)**, and Venn diagrams of taxonomic overlap of core microbiomes **(C,F)** are shown for bioswales, green roofs and non-engineered sites (tree pits and park lawns). Core microbiomes were defined as the set of ASVs present in at least half the samples in a given group, with a mean abundance of at least 10 across all sites. Differences in mean phylogenetic diversity values were evaluated with Kruskal–Wallis and Wilcoxon tests. ANOSIM tests for clustering were significant (*p* < 0.001) for all groups shown. *p < 0.05, **p < 0.01, ***p < 0.001.

**FIGURE 3:**
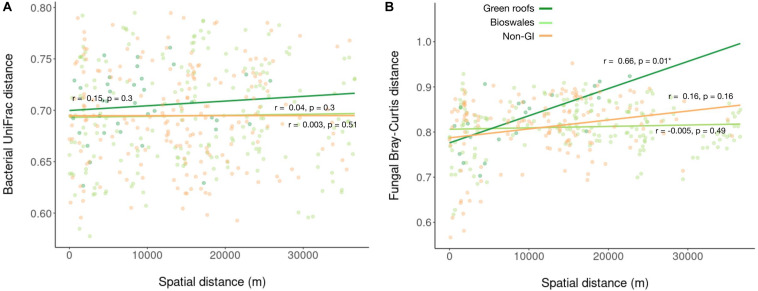
Linear regression of bacterial **(A)** and fungal **(B)** pairwise Bray–Curtis distances against pairwise spatial distances.

Following demultiplexing and quality filtering, Miseq sequencing of bacterial 16S amplicons yielded 2,110,677 reads assigned to 18,422 ASVs, with an average of 16,236 reads across the 130 samples. Bacterial phylogenetic diversity was highest in green infrastructure (GI) soil sites (Figure 2A). Green roofs and streetside swales were the most diverse, followed by right-of-way swales; all three GI soil types were more diverse than conventional GI soils, i.e., tree pits and park lawns (not shown). Tree pits and park lawns demonstrated broadly similar microbial profiles relative to other samples in the study, with no distinct clustering based on either Bray–Curtis or UniFrac distances, and were therefore grouped as non-engineered GI soils. Bioswales, green roofs and non-engineered GI soils were all compositionally distinct (Figure 2B). These differences were in part driven by the presence of unique variants in green infrastructure communities (123 core ASVs unique to green roofs and bioswales combined, compared to 20 in parks and tree pits) (Figure 2C). Bacterial communities were also significantly clustered when grouped by engineered GI versus non-engineered GI soils, by rooftop versus ground level, and by individual soil type (ANOSIM *p* < 0.001), but not by borough. Differences in bacterial community composition were not explained by geographic distance among sites within any set of samples (Figure 3).

**FIGURE 4:**
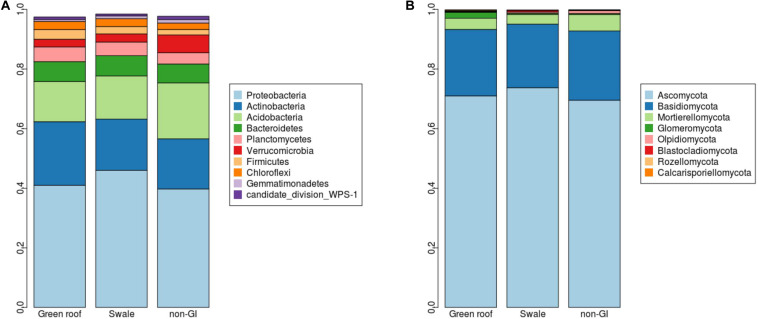
Relative abundances (y-axes) for **(A)** bacterial and **(B)** fungal phyla detected green infrastructure types separated by green roofs, swales, and conventional (conventional, non-engineered) installations.

Marked differences between engineered and non-engineered GI sites at the level of 16S sequence variants (i.e., at or near the species level) were not evident at higher taxonomic levels. Communities in all sites had broadly similar phylum-level composition, with just four phyla comprising over 80% of all communities: Proteobacteria, Actinobacteria, Acidobacteria, and Bacteriodetes (Figure 4). Verrucomicrobia was the most differentiated phylum, with greatest abundance in conventional GI sites.

**FIGURE 5:**
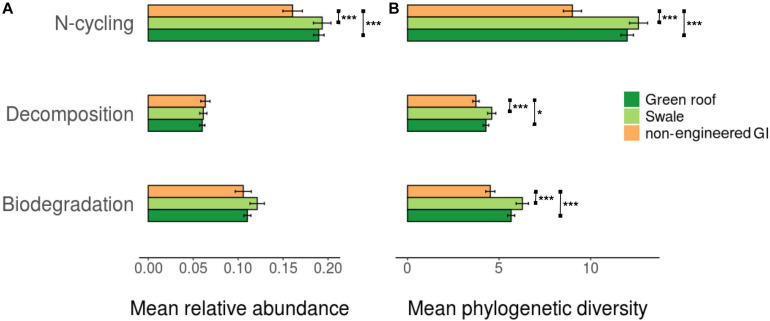
Average relative abundance **(A)** and phylogenetic diversity **(B)** for trait-associated bacterial variants (*p < 0.05, **p < 0.01, ***p < 0.001).

**FIGURE 6:**
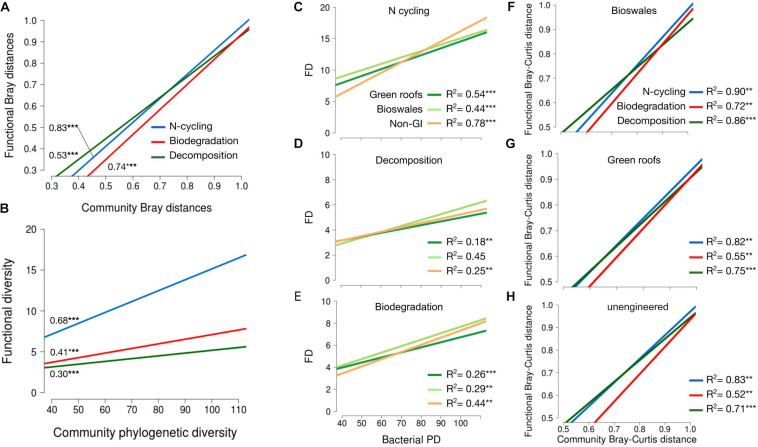
**(A)** Linear regression of pairwise Bray–curtis distances for trait-associated variants against community pairwise Bray–Curtis distances. *R*^2^ values shown denoted with significance as indicated. **(B)** Linear regression of functional diversity (phylogenetic diversity of trait-associated variants) against community phylogenetic diversity. *R*^2^ values shown denoted with significance as indicated. **(C–E)** Functional versus phylogenetic diversity as in plot **(B)**, but broken down by habitat type for nitrogen cycling **(C)**, decomposition **(D)** and biodegradation **(E)** traits. Linear regression of pairwise beta-diversity differences for trait-associated variants versus community distances, shown separately for bioswales **(F)**, green roofs **(G)**, and non-engineered sites **(H)**. All data shown refer to bacterial communities. *p < 0.05, **p < 0.01, ***p < 0.001.

To provide an indication of functional diversity, ASVs were compared to lists of genera compiled for taxa associated with any of three functional categories: N-cycling processes, biodegradation and bioremediation, and decomposition. Extracting these groups resulted in sets of 2,083, 869, and 652 variants, respectively. N-cycling and biodegrading bacteria were more abundant in green roof and bioswale communities compared to non-engineered GI sites (Figure 5). Differences in taxa associated with N-cycling, biodegradation and decomposition were predictable based on the degree of divergence among total communities (Figure 6).

**FIGURE 7:**
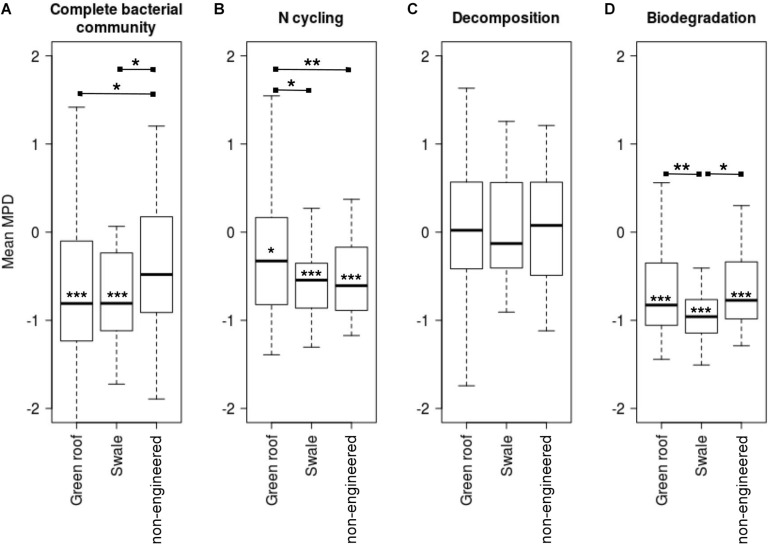
Mean pairwise distances (MPD) for total bacterial communities **(A)**, and variants related to sets of nitrogen cycling **(B)**, decomposition **(C)**, and biodegradation **(D)** traits. Asterisks on mean bars denote significance values for *t*-tests comparing means to zero, which signifies the null expectation for local phylogenetic diversity given the species pool (mean MPD significantly below zero signifies phylogenetic clustering). Asterisks between groups indicate significance of *t*-tests for pairwise differences of group mean MPD values.

Mean pairwise distances for all pairs of variants were calculated to assess the phylogenetic distribution of bacterial communities relative to a null model. Communities in green roofs and bioswales were more phylogenetically clustered than expected by chance, given the detected local species pool (Figure 7). Communities of taxa associated with nitrogen cycling and biodegradation were clustered across all soil types, though decomposer communities were relatively evenly distributed.

### Fungi

Sequencing of ITS amplicons resulted in 2,933,063 reads that clustered into 7,448 ASVs, of which 10,262 were identified as fungal, distributed across 102 samples. Bioswale fungal communities were significantly more diverse than those from green roofs and non-engineered GI soils, which had similar Shannon diversity (Figure 2D). Although not as diverse as bioswales, green roof fungal communities were more distinct, clustering apart from other soil types based on Bray–Curtis community distances (Figure 2E). Bioswales were more similar to conventional GI sites, though they were still significantly differentiated (ANOSIM *R* = 0.11, *p* = 0.002), with the greatest share of unique ASVs (Figure 2F). Unlike with bacteria, geographic distance appears to partly explain community dissimilarities for green roofs (Figure 3B). Geographic distance did not explain differentiation among bioswale or non-engineered GI communities.

**FIGURE 8:**
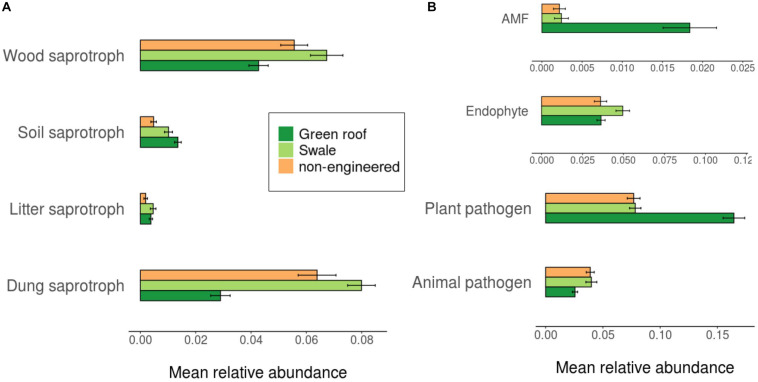
Average relative abundance of trait-associated fungal variants.

Ascomycota fungi were dominant in all communities, followed by Basidiomycota. Differentiation of green roof fungal communities was not evident at the phylum level, where they appeared broadly similar to bioswales (Figure 4). FUNGuild analysis linked 2,433 fungal variants to guilds at confidence level “Probable” or “Highly probable.” Several functional guilds had notable representation in fungal communities, including saprotrophs (1,228 variants, 20.1% mean relative abundance across all sites), plant and animal pathogens (694, 14.2%) endophytes (170, 3.9%), and arbuscular mycorrhizae (184, 1.1%). The large majority of saprotrophs were associated with wood and dung, followed by soil and then litter (Figure 8).

## Discussion

Our results demonstrate that engineered GI soils sustain compositionally and functionally distinct microbial communities differentiated from those in non-engineered, conventional GI sites park and tree pit soils. GI bioswales and green roofs had particularly high microbial biodiversity, with greater representation of bacterial and fungal lineages than conventional GI sites, including taxa associated with key ecosystem services involving nitrogen cycling, biodegradation, and decomposition. Patterns of functional and community diversity suggest that unidentified elements of GI designs—probably related to factors like Technosol composition, substrate loads in stormwater runoff or plant community composition—drive habitat-specific community assembly processes, pointing to both the potential practicality and value of incorporating the management of urban soil microbial resources into GI programs.

### Urban Soil Microbial Diversity and Biogeography

This study provides further evidence that urban soils can be comparable in microbial richness and diversity to less anthropogenically-dominated biomes (Ramirez et al., 2014). Bioswales and green roofs, respectively, contained the greatest phylogenetic diversity and richness of bacteria, and bioswales had the most diverse fungal communities, indicating that GI installations are effective reservoirs of microbial biodiversity at both rooftop and ground level. Bioswales appeared to be among the most microbially diverse urban soils studied to date, given previous results showing that tree pits, medians and parks all had broadly similar bacterial alpha diversity levels (Reese et al., 2016; Gill et al., 2017). GI soils were not only relatively diverse, but also compositionally distinct from non-engineered GI communities. Fungi and bacteria were structured by habitat type, with differentiated communities in green roofs, bioswales, and non-engineered GI sites. Surprisingly, bacterial communities in bioswales and green roofs were more similar to each other than to tree pits or parks, despite shared status as ground-level sites with similar exposure to stressors like pedestrians, pets, and vehicular pollution that are less likely to affect rooftop communities. The fact that rooftop and GI street-level communities shared similarities despite starkly different environments suggests that one or more GI design elements are contributing to the biogeography of NYC soil microbes. This may explain the relatively high number of core taxa shared exclusively among bioswales and green roofs compared to the 10 that appeared in non-engineered GI soils and either green roofs or bioswales, but not both (Figure 2).

### Microbial Community Assembly

Analysis of distance-decay relationships, habitat-specific phylogenetic clustering patterns, and relationships between community diversity and trait-associated functional diversity were all consistent with the hypothesis that diversity and compositional differences in GI soil communities are driven by elements of GI designs rather than stochastic processes. Mechanistically, this may include environmental filtering, i.e., the exclusion of lineages poorly adapted to abiotic conditions in conventional GI sites. Environmental filtering is difficult to distinguish from forces rooted in species interactions (Kraft et al., 2015; Cadotte and Tucker, 2017), and we acknowledge that GI specifications may also lead to differentiation through competition, symbiosis, or other biotic interactions.

Several alternate explanations to GI-specific biotic and abiotic filtering are possible. For example, history might explain the similarity of bioswale and green roof microbiomes if they are derived from commonly sourced soil media. This is probably not the case, however, since GI soils, though not sterilized, are sourced from multiple contractors who obtain soils from wherever they wish as long as GI specifications are met (Nandan Shetty, personal communications). Age could also drive differences if GI sites are newer than the conventional GI habitats in this study—however, NYC has built many tree pits in recent years as part of its Million Trees program^2^, which was concurrent with much of the active phase of GI construction. Given the exposed nature of GI sites and many opportunities for short-distance dispersal from many conventional GI soil sources, it is unlikely that age is the primary reason for similarity of GI microbial communities. Still, as GI installations mature it will be worthwhile to examine GI soil microbial communities longitudinally to understand how installation age affects microbial diversity. As discussed below, history and age would also not necessarily predict the differences in functional taxa found here, whereas likely explanatory factors related to stormwater inputs and plant communities are directly impacted by GI designs.

#### Distance-Decay Relationships

Under an alternative scenario of neutral community assembly, similarity among urban soil sites would be a function of distance, assuming that dispersal is present but limited (Zhou and Ning, 2017). Dispersal limits are indeed likely among these sites—though many bacteria and fungal spores can readily disperse (Barberan et al., 2015), sub-surface microorganisms have reduced mobility (Lindqvist and Bengtsson, 1991) and sites were isolated by impervious surfaces and elevation. Under deterministic community assembly, site-specific biotic and/or abiotic factors select among an available species pool (formed in part through dispersal), leading to greater community similarity in environmentally similar sites rather than geographically proximate sites. This leads to the prediction that different habitat types would show different distance-decay relationships as migrating taxa meet different selective conditions in green roofs, bioswales and conventional GI sites.

We instead found that dissimilarity among all microbial communities, except fungi in green roofs, was not related to spatial distance. Selection and drift, in combination with limited dispersal, generally lead to strong distance-decay relationships, with strong dispersal acting in the opposite direction (Hanson et al., 2012). Ecological selection, however, tends to contribute to distance-decay relationships only to the extent that environmental or other selective agents are spatially autocorrelated. This would not be expected for GI-related design features that control for many factors likely to contribute to filtering—pH, nutrient levels, soil composition, protection from compaction, managed plant communities, stormwater input for bioswales (Deeb et al., 2018). We accordingly interpret the lack of distance-decay relationships in combination with habitat-specific clustering as indicative of environmental filtering, as well as the absence of severe dispersal limits for urban soil bacteria. We note that this does not exclude the operation of neutral processes, but rather suggests that different urban microhabitats impose unique biotic and/or abiotic filters on the available regional species pool, resulting in differentiated microbial communities (Jeraldo et al., 2012).

Phylogenetic clustering patterns were also consistent with filtering, which may act to exclude poorly adapted lineages, and simultaneously favor other clades of ecologically and phylogenetically related variants with increased fitness in local environments (Pavoine and Bonsall, 2011). Bacterial communities in bioswales and green roofs were both more phylogenetically clustered and more phylogenetically diverse than non-engineered sites, an apparent contradiction that can be explained by filtering acting over different phylogenetic scales (Nemergut et al., 2013). Environmental filtering contributes to clustering to the extent that environmentally-favored traits are phylogenetically conserved, reducing phylogenetic diversity at broad scales (O’Dwyer et al., 2012). Favored lineages may simultaneously retain high diversity at lower phylogenetic levels as a result of greater resource availability or other site-specific factors. Mechanistically, one explanation involves stabilizing niche differences in engineered soils. If there is deep phylogenetic coherence to nitrogen cycling and decomposition traits, as has been seen previously for various microbial functions (Philippot et al., 2010), then strong clustering in bioswales may be driven by an increase of available niche space for lineages well-adapted to resources associated with stormwater, or associated with GI-specified plant communities. Data on soil physiochemistry, micro-climate, and plant composition were not available for this study, and future surveys and experimental studies including these parameters will be critical in delineating the relative contributions of history and specific biotic and abiotic deterministic factors in shaping urban microbial community assembly in GI and other human-modified environments.

### Diversity of Trait-Associated Microbes

We hypothesized that the GI-specific community differentiation would be accompanied by differences in taxa linked to functional traits, and we found that GI soils indeed contained bacterial and fungal assemblages consistent with elevated functional potential and redundancy. For bacteria, general patterns of functional diversity roughly mirrored community-level phylogenetic diversity—engineered GI soils had greater relative abundance and trait-specific phylogenetic diversity than conventional GI sites for nitrogen cycling and biodegradation, and greater phylogenetic diversity of decomposer taxa. Fungi also exhibited considerable functional diversity, pointing to the presence of diverse sources of metabolic activity in urban soils. Unlike for bacteria, however, fungal trait-associated composition did not differ clearly across engineered versus non-engineered sites, with the exception of greater proportions of dung and litter saprotrophs in bioswales.

Trait divergence in all three sets of bacterial functions predicted community divergence, indicating that compositional differences are linked to functional differences between communities in different site types. Differences among more similar communities tended to by driven by differentiation in decomposer taxa, but that relationship shifted as community divergence increased, with nitrogen cycling and biodegradation contributing relatively more to total community differences. Alpha diversity of all three traits was also a significant predictor of total community phylogenetic diversity, pointing to the importance of trait-based differences among urban soil patches. Interestingly, the functional-phylogenetic diversity relationship was different in GI and conventional GI communities. Functional diversity predicted phylogenetic diversity for all three trait groups and the relationship tended to be strongest in non-engineered soils. This suggests differences in niche structure, with stabilizing niche differences in bioswales maintaining consistent levels of trait-associated taxa, while restricted niche space in non-engineered soils with lower phylogenetic diversity appears to be resulting in competitive exclusion of many of those same variants. Stochastic community assembly may be a factor in the fraction of phylogenetic diversity that varies among sites of the same type, e.g., to explain differences in alpha diversity among communities that contain consistent levels of functional diversity.

Functional diversity patterns were consistent with the hypothesis that components of bioswale bacterial communities are responding metabolically to substrates contained in stormwater runoff, which includes both nitrogen and multiple classes of common urban chemical pollutants (Gasperi et al., 2008). If these substrates, which are unlikely to reach tree pits, parks or green roofs, select for taxa that can metabolize them, we would expect community differentiation to be accompanied by differentiation in trait associated taxa. This was observed for nitrogen cycling taxa as well as bacteria linked to one or more of over 200 pathways targeting 50 classes of compounds, including hydrocarbons, pesticides, phenolics, and other contaminants—both functional groups were most diverse in bioswale soils designed to intake stormwater runoff. Bacterial variants associated with nitrogen cycling and biodegradation were also phylogenetically clustered, and the largest differences in beta-diversity were associated with compositional divergence of trait-associated taxa. These patterns also support a selective role for environmental factors related to substrates of nitrogen cycling and biodegradation pathways.

A key question is whether the presence of functional groups of taxa implies that related substrates are being metabolized at higher rates than in less diverse soils, and whether ecological functions underlying ecosystem services are being performed (Rocca et al., 2015). Mesocosm experiments have indeed shown that declines in functional diversity lead to reduced rates of related ecosystem processes, even when controlling for functional gene and substrate abundances (Trivedi et al., 2019). If GI soils host taxa capable of breaking down contaminants before they either leach through soil or enter surrounding waterways, that would represent a valuable co-benefit of GI installations that could be optimized in its own right, similar to how GI designs—including the identity of plant communities—are presently optimized for stormwater infiltration and retention. For nutrient cycling, diversity effects are most significant at lower taxonomic levels, and composition rather than taxonomic diversity is most important for ecosystem functioning (Bardgett and van der Putten, 2014). The compositionally differentiated, comparatively diverse communities of bacterial variants linked to nitrogen cycling traits in bioswale and green roof soils are consistent with increased performance of these processes, but future experimental studies will be needed to verify how shifts in functional diversity affect process rates in GI soils.

The high patches of net primary productivity provided by urban green spaces surrounded by impermeable surfaces is a noted driver of biogeographic patterns in macroorganisms (Shochat et al., 2006; Ossola et al., 2016), and our results point to the possible importance of plant communities in shaping microbial biogeography. GI soils tend to have greater diversity of plant communities due to ecological management than tree pits and parks, which tend to be relatively bare of vegetation or planted with grass monocultures, respectively. Accordingly, we observed greater abundance and diversity of endophytic fungi in bioswales, as well as a roughly sevenfold increase in arbuscular mycorrhizal fungi in green roofs. The latter finding is consistent with the presence of Glomeromycota associated with a variety of plants found previously on green roofs (McGuire et al., 2013).

### Summary

Our results suggest that anthropogenically-modified, fragmented soils in urban ecosystems contain considerable biodiversity of microbial communities that are compositionally distinct across different types of green infrastructure. While this trend was suggested by previous studies, this was the first study to simultaneously analyze multiple green infrastructure types, including engineered and non-engineered soil. Ecologically engineered green infrastructure soils in particular sustain differentiated, comparatively diverse microbial communities with elevated functional diversity of taxa linked to several ecosystem services. Based on differences in beta diversity, distance-dispersal relationships, and associations between community structure and functional diversity, we conclude that habitat-specific environmental filtering is likely an important factor in structuring urban microbial communities. Environmental differences rooted in GI designs appear to be driving community differentiation, resulting in bioswale and green roof communities with distinct profiles of taxa.

The functions examined in this study explain only a portion of beta diversity differences between urban soil habitats. Future studies should account for edaphic and other environmental gradients, stormwater nutrient and pollutant loads, plant composition, and process rates for microbially provisioned ecological functions to more precisely identify the drivers of microbial composition and function. Further efforts to understand how green infrastructure and other urban land management approaches shape microbial communities will promote increased inclusion of microbial processes in ecological engineering efforts. Since urbanization is projected to continue increasing, incorporation of soil biological processes driven by microbes will be essential for ecological conservation and management efforts that include the expansion of green infrastructure networks in urban environments (Guilland et al., 2018).

## Data Availability Statement

Data are now publicly available through the NCBI Sequence Read Archive (Accession# PRJNA633213 ID# 633213).

## Author Contributions

AG, KM, JS, and MP designed the study. KP and AG conducted soil sampling and processing. AG analyzed the data. AG, KP, and KM wrote the manuscript.

## Conflict of Interest

The authors declare that the research was conducted in the absence of any commercial or financial relationships that could be construed as a potential conflict of interest.

## Footnotes

1 github.com/grenaud/deML

2 milliontreesnyc.org
